# Comparison of neck length, relative neck length and height with incidence of cervical spondylosis

**DOI:** 10.12669/pjms.36.2.832

**Published:** 2020

**Authors:** Syeda Bushra Ahmed, Aisha Qamar, Muhammad Imram, Muhammad Faisal Fahim

**Affiliations:** 1 Dr. Syeda Bushra Ahmed, MBBS, M.Phil Scholar Anatomy; 2 Prof. Dr. Aisha Qamar, MBBS, M.Phil Anatomy. Department of Anatomy, Bahria University Medical and Dental College, DHA Phase-II, Karachi, Pakistan; 3 Dr. Muhammad Imran, MBBS, FCPS. HOD Department of Radiology, Patel Hospital, Karachi, Pakistan; 4 Muhammad Faisal Fahim, M.Sc (Statistics), Researcher and Consultant Statistician, Bahria University Medical and Dental College, DHA Phase-II, Karachi, Pakistan

**Keywords:** Cervical spondylosis, Height, Kellgren Lawrence grade scale, Radiography, Neck length, Relative neck length

## Abstract

**Objective:**

To compare the neck length, relative neck length and height between patients with cervical spondylosis and healthy subjects.

**Methods:**

This case control study was conducted at Patel hospital, Karachi after the ethical approval of Bahria University Medical and Dental College (BUMDC) and Patel hospital from September 2018 - February 2019. It enrolled eighty eight cases of cervical spondylosis and eighty eight healthy subjects. Radiographs were taken in the lateral view and neck length was measured as the distance from external occipital protuberance to seventh cervical vertebra spinous process. Then relative neck length was measured by dividing the neck length with height and multiplying it by 100. The Kellgren Lawrence grade scale was used to assess the severity of cervical spondylosis.

**Results:**

A total of 176 participants were analyzed. It was found that the height remains the significant determinant. The comparison of cases with control group was done using independent T-test which showed that the cases were significantly shorter than controls with a p-value < 0.05. The other variables such as neck length, and relative neck length were insignificant.

**Conclusion:**

Short height can be considered as a risk factor for cervical spondylosis. Short-statured individuals should be counseled to adopt measures for the prevention of cervical spondylosis.

## INTRODUCTION

Worldwide, spinal diseases are the most common disorders affecting the cervical and lumbar spine. They are the degenerative changes occurring in relation with age, gender, occupation and lifestyle. The neck pain is one of the most frequent presenting complaints caused by uneven distribution of mechanical load at spine.^1^

Cervical spondylosis is defined as “the osteoarthritic degeneration of cervical spine components such as uncovertebral, facet joints and intervertebral discs causing sensory and motor dysfunctions”. The pathogenesis of cervical spondylosis involves the formation of osteophytes, disc herniation, and hypertrophy of ligaments.^2^

Many factors are responsible for causing cervical spondylosis such as forward head posture^3^,^4^, neck strain, stress, depression, sports, and occupational activities. Symptoms include pain in the neck and arms as well as numbness in the arms and fingers. The prevalence of neck pain is around 23.1% and incidence peaks up about 21.3% in the high-risk population of office and computer workers.^5^

It is most common in people aged over 55 years. It is also getting prevalent in young generation which is a major health concern. Some atypical manifestations have also been reported which include insomnia, headache, vertigo, nausea, abdominal discomfort, palpitation, amnesia, blurred vision, and tinnitus.^6^

It presents as three syndromes that are, axial neck pain, cervical radiculopathy, and cervical myelopathy. Axial neck pain is the universal presentation due to improper posture and dysfunction in ligamentous or bony elements of the cervical spine. Cervical radiculopathy is due to impingement of intervertebral foramen while cervical myelopathy is due to narrowing of cervical spinal canal.^7^

Loss of lordotic curve also affects the cervical range of motion.^8^ By the age of 60 years, all elderly individuals show spondylotic changes in the radiological investigation. Surgery is not the first choice in asymptomatic elderly patients, and conservative therapeutic strategies should be implemented as the mainstay of treatment.^9^

A study reported that in individuals less than 30 years of age, gender, work hours and same work posture were the associated factors. Housework intensity was responsible in the age group of 30 to 45 years. Walking displayed a protective role and should be considered as a preventive measure of cervical spondylosis.^10^

A recent Japanese study demonstrated an increase in the spinal canal diameter, height, body weight and arm span predicting decreased cases in the future owing to the improved nutritional and environmental factors.^11^ Keeping all these factors in consideration, this study was undertaken to determine the association of cervical spondylosis with respect to neck length, relative neck length and height in our population.

## METHODS

The present study was conducted at the Patel Hospital Karachi, after obtaining ethical approval from Bahria University Medical and Dental College (BUMDC) (Ref. No. ERC 45/2018) and Patel Hospital (Approval No. 57) from September 2018 – February 2019. The subjects were enrolled from orthopedic OPD. It was a case-control study including patients between the ages of 25 and 75 years. Eighty eight diagnosed cases of cervical spondylosis and eighty eight healthy controls were selected using convenient sampling technique from hospital premises. Written informed consent was obtained from both patients and controls. Any patient with cervical tumor, cervical rib, and systemic diseases of bones, thyroid, parathyroid disorders, pregnancy, trauma, and cases with surgery of cervical spine were excluded from the study.

**Fig.1:**
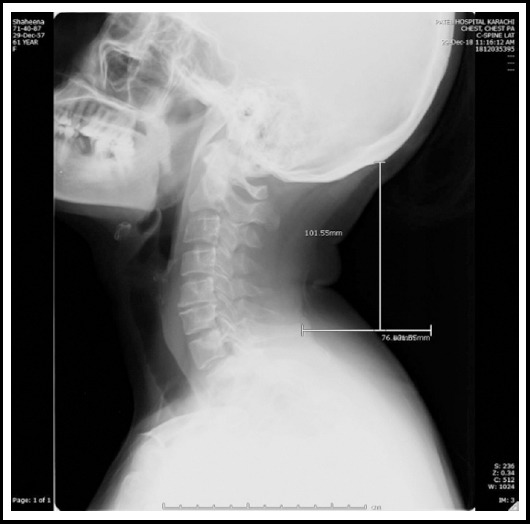
X-ray cervical spine (lateral view) demonstrating radiological parameters for measuring neck length on Synapse.

A thorough history and detailed examination of the selected cases and controls was carried out in the orthopedic OPD, followed by a radiograph of cervical spine in the lateral view under supervision of the senior radiologist. The weight and height were also measured with the help of body weight and health scale model no ZT-120. The subject evaluation form was filled by the principal investigator asking the questions related to complaints of neck pain, occupation and usage of electronic devices etc. The participants were asked to stand upright with the neck in neutral position and to depress the shoulders. The neck length was measured by measuring the perpendicular distance between the external occipital protuberance and the tip of the seventh cervical vertebra using a software Synapse (Fujifilm Medical Systems, Tokyo, Japan) (Fig.1). It was cross-checked by the senior radiologist. The relative neck length^12^ was calculated by dividing the neck length with the total height of the individual. The formula is mentioned below:


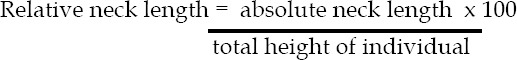


The Data obtained was entered in the subject evaluation form and analyzed using software SPSS version 23.

## RESULTS

A total of 176 adults (73 males and 103 females) between ages 25 and 75 years participated in the study. The mean neck length in cases and controls was 104.15±18.9 mm and 106.98±19.0 respectively. The relative neck length in cases and controls was 6.90±0.89mm and 6.93±0.87mm respectively. The mean weight in cases and controls was 69.62±11.2kg and 66.64±12.44kg. The height in cases and controls was 163.31±9.88cm and 166.22±9.07cm respectively, with a significant p-Value (Table-I). The mean neck length was significantly shorter in females as compared to males. The height in males was 168.81±8.42cm and in females it was 159.14±8.88cm which was highly significant. The mean weight in females was significantly less as compared to males (Table-II). The comparison between cases and control group was done using Independent T-test and it was found that cases were significantly shorter than controls. The other variables like neck length, relative neck length and weight demonstrated insignificant differences in between cases and controls.

**Table-I:** Comparison of Neck length, relative neck length and height between cases and controls (N=176).

Variables	Cases (N=88) Mean±S.D	Controls (N=88) Mean±S.D	P-value
Neck length (mm)	104.15±18.9	106.98±19.0	0.326
Relative neck length (mm)	6.90±0.89	6.93±0.87	0.823
Height (cm)	163.31±9.88	166.22±9.07	0.044*
Weight (kg)	69.62±11.2	66.64±12.44	0.097

P value <0.05 is considered statistically significant, marked as

* Test applied: Independent T-test.

**Table-II:** Comparison of Neck length, relative neck length and height within cases between males and females (N=88).

Variables	Male (N=38) Mean±S.D	Female (N=50) Mean±S.D	P-value
Neck length (mm)	109.08±16.94	100.42±19.78	0.033*
Relative neck length (mm)	7.01±0.90	6.82±0.90	0.317
Height (cm)	168.81±8.42	159.14±8.88	0.000*
Weight (kg)	73.94±11.93	66.34±9.49	0.001*

P-value <0.05 is considered statistically significant, marked as

* Test applied: Independent T-test.

## DISCUSSION

Neck length has been defined as the midline and lateral neck length. A midline neck length (MNL) is the distance between the upper part of the hyoid bone to the jugular notch and lateral neck length (LNL) is the distance from mandibular angle to the middle portion of ipsilateral clavicle. They were measured in relation to the height and correlated with sleep and cardiovascular risk factors. According to a study, men with shorter LNL height ratio were suffering from metabolic syndrome, whereas women with short MNL height ratio were found to be associated with snoring and cardiovascular risk factors.^13^

In another study neck length was defined as cricosternal distance; that is, distance between cricoid cartilage and sternal notch.^14^ Another study tried to standardize the age-dependent interrelationship of neck length with other growth parameters. They measured neck length between inion and C7 spinous process in a neutral position with standing and sitting height in children up to mid-adolescents as percentiles and ratios. These measurements were statistically significant and provided values for quick assessment of short neck in Indian population.^12^

This study is first of its kind in Pakistan, comparing the neck length, relative neck length and total height in relation to cervical spondylosis between cases and controls. It reports the measurements of neck length, relative neck length, and height in our population. It also gives an estimate of the average height in male and female population of Karachi.

The neck length was measured as the distance from external occipital protuberance to the C7 spinous process. A study reported that cases with cervical spondylosis demonstrated short neck length.^15^ This was in agreement to the present study, where cases showed decreased neck length as compared to controls, although difference was not significant. This finding could be attributed to racial differences. The present study also determined a significant decrease in neck length in females as compared to the males which demonstrated that neck length was directly proportional to the height. The height and weight was significantly higher in the men as compared to the women in another study.^16^

Height is defined as short or tall as the upper and lower limit of normal curve as 3^rd^ and 97^th^ percentile.^17^ In a study, individuals with shorter height displayed protective role against cardiovascular diseases.^18^ Another study explored association of microalbuminuria with height and weight and found out that taller height and lower weight subjects had increased incidence of microalbuminuria.^19^

The present study revealed that incidence of cervical spondylosis was more in short statured subjects. Thus increase in height serves as a protective factor against occurrence of cervical spondylosis. This was supported by a study which demonstrated that cervical spinal canal dimensions had a positive relationship with body height.^20^ Singh et al. (2014)^21^ demonstrated that mean height of group with radiculopathy and myelopathy was 156.58 ± 8.84 cm, that is shorter than the mean height of control group (159.54 ± 8.17 cm), again emphasizing a protective effect of tall height in cervical spondylosis. This is in accordance to the present study, which showed a mean height of 163.31±9.88cm in cases and 166.22±9.07cm in controls.

In 2016 the Non-Communicable Diseases Risk factor Collaboration NCD-RisC surveyed different countries and reported the tallest height over 182.5cm in the Netherlands and shortest in the Guatemala with a height range of 135.8-144.8cm.^22^ However, in the present study the tallest height was 176 cm and shortest was 154cm. The average height in males was 168.81±8.42cm and in females it was found to be 159.14±8.88cm.

## CONCLUSION

According to the present study, individuals with short height were more prone to cervical spondylosis delineating short stature as a risk factor. Short-statured individuals should focus on maintaining a healthy lifestyle, such as exercise, correct work posture, and maintenance of body weight within normal range to prevent cervical spondylosis. The estimate of average height in our population for the risk of cervical spondylosis can also be considered by clinicians to identify patients at risk and implement appropriate measures.

## RECOMMENDATIONS

The study should be done in multiple tertiary care hospitals to correlate other patient variables to identify the risk factors and prevalence of disease in our population.

### Author’s Contribution:

**SBA** designed the study, did data collection, compilation and write up of manuscript, is responsible for integrity of research.

**AQ** did critical review and editing of manuscript.

**MI** helped in data collection and final approval of manuscript.

**MFF** did statistical analysis.
